# Examining psychological correlates of vaccine hesitancy: a comparative study between the US and Israel

**DOI:** 10.3389/fpubh.2024.1480419

**Published:** 2025-01-03

**Authors:** Nicolle Simonovic, Anat Gesser-Edelsburg, Jennifer M. Taber

**Affiliations:** ^1^The Health and Risk Communication Lab, School of Public Health, University of Haifa, Haifa, Israel; ^2^Department of Psychological Sciences, Kent State University, Kent, OH, United States

**Keywords:** vaccine hesitancy, health behavior, risk perception, emotions, ambiguity, intentions

## Abstract

It is important to identify psychological correlates of vaccine hesitancy, including among people not from the United States (U.S.). College students were recruited between March–June 2023 in the US (*n* = 330, *M*_age_ = 20.21, 79.5% female) and in Israel (*n* = 204, *M*_age_ = 23.45, 92.6% female) to complete a cross-sectional survey on vaccine attitudes, emotions, and behavior. A 2 (Nation: US, Israel) × 2 (Vaccine Status: Vaccinated, Unvaccinated) factorial design was used. Individual ANCOVAS controlling for sociodemographic factors were conducted to test main effects of nation and vaccine status, and their interaction, across various psychological correlates of health behavior. Consistent with hypotheses, unvaccinated (vs. vaccinated) individuals reported higher perceived ambiguity, reactance, and anger as well as perceived lower susceptibility, severity, worry, and intentions to vaccinate. Unvaccinated (vs. vaccinated) individuals also reported lower positive emotion. Contrary to hypotheses, unvaccinated individuals reported greater fear. Israeli (vs. American) participants reported higher perceived ambiguity, worry, fear, and anger, as well as lower perceived susceptibility. Vaccinated Americans reported higher intentions to vaccinate again in the future (*M* = 2.89, SE = 0.08) compared to vaccinated Israelis (*M* = 2.36, SE = 0.08). However, unvaccinated Americans reported lower intentions to vaccinate (*M* = 1.80, SE = 0.15) than unvaccinated Israelis (*M* = 1.95, SE = 0.21). Findings provide insight into correlates to target for vaccine promotion and emphasize the need for cultural tailoring.

## Introduction

Prior to the onset of the COVID-19 pandemic, vaccines prevented approximately 4–5 million deaths a year (1). More recent estimates indicate that at least 14.4 million deaths were prevented in 1 year by the COVID-19 vaccine (2). Additional deaths across all vaccine-preventable illnesses (e.g., Measles, Pertussis, and COVID-19) could be averted if vaccination rates increased. However, vaccine hesitancy—the delay or refusal to receive available vaccines (3)—serves as a barrier to vaccination. The purpose of the present research is to examine psychological correlates of vaccine hesitancy in a cross-cultural sample of Americans and Israelis.

Various behavioral theories provide insight into why people do not engage in protective health behavior, such as vaccination. According to the Health Belief Model (4), Theory of Planned Behavior (5), Extended Parallel Processing Model (71), the Appraisal Tendency Framework (6–8), Reactance Theory (9), PRECEDE PROCEED Model (10), and Transtheoretical Model of Behavior Change (11), several cognitive, affective, and behavioral constructs are at play in health decisions. Some of the many constructs involved in health decisions include risk perceptions, emotions, knowledge, reactance, and intentions. Other psychological variables relevant to health behavior that are often overlooked in major theories of health behavior include ambiguity and positive emotions. In the next section, we provide an overview of each of these variables, including a brief description and selected research pertaining to vaccination.

### Psychological correlates of vaccine hesitancy

*Risk perceptions* refers to subjective beliefs pertaining to an outcome, such as illness. Individuals can hold beliefs about: their *susceptibility* or likelihood of contracting an illness, how *severe* or harmful the illness may be, as well as how *worried* they are about the illness (12, 13). Higher perceptions of risk about illness often predict greater vaccine use (14). However, higher perceptions of risk about vaccination, rather than the illness itself, can promote vaccine hesitancy or refusal (15).

In addition to risk perceptions, *perceived ambiguity* is another important psychological determinant of vaccine hesitancy. Ambiguity refers to the experience of uncertainty that specifically arises from perceiving information to be unreliable, low in credibility, or inadequate, which is often the case when information is conflicting, imprecise, or incomplete (16, 17). Perceiving ambiguity about vaccination (e.g., about side effects or the efficacy of the vaccine) can lead to avoidance of vaccination (18–20).

Next, *emotions* are important in understanding health behavior, as health behaviors likely occur within an emotional context (21). For example, individuals may deny or refuse vaccination because they experience *fear* about potential side effects of vaccination (22, 23). *Anger* is another emotion that may lead to hesitation to vaccinate (24, 25). Indeed, anger may activate schemas of distrust in others and lead to greater mistrust in the medical system (26). As for *positive emotions* (e.g., happiness, relaxation, etc.), more research is needed to understand the relationship between positive emotions and health behavior (27). However, related to vaccination, researchers have demonstrated that vaccine hesitant individuals are less likely to experience positive emotions compared to their counterparts who are more inclined to vaccinate (28). Further, there are various positive experiences (affective and cognitive) that may contribute to positive emotion—such as experiences of hope, optimism, trust, and altruism—that can, in turn, promote vaccination (25, 29–33). Indeed, vaccines may offer hope and optimism, such as in the case of a cancer vaccine or an AIDS vaccine that can improve survival outcomes and reduce transmission (34, 35). Further, fostering trust and altruistic motives may increase willingness to vaccinate (36, 37). Future cross-cultural research on vaccination behavior should assess these factors. Another factor relevant to health behavior engagement is knowledge. In particular, those who have a better understanding of what actually presents a health risk are more likely to engage in behaviors aimed at avoiding that health risk. Indeed, adults who are more knowledgeable about preventive health behaviors (e.g., handwashing or avoiding crowds) are more likely to engage in these behaviors during a pandemic (38, 39). Further, researchers have demonstrated a relationship between higher knowledge about vaccination and a positive attitude toward vaccination (40).

Finally, *intentions* are an especially important component of health behavior because intentions to engage in a behavior, or lack thereof, often precede the decision to engage in health behavior (41). However, experiencing *reactance*—a motivational state in which people attempt to re-establish autonomy in the face of feeling like their freedom is threatened (9)—can decrease intentions to vaccinate (42).

Importantly, no singular health behavior theory provides a comprehensive model of all psychological factors relevant to vaccination, and including all variables across health behavior theories would be beyond the scope of the present research. With our aim to examine psychological correlates of vaccine hesitancy, including some afforded less attention in major theories of health behavior, we selected correlates for inclusion based on variables that overlap across many different theories of health behavior (e.g., risk perception, knowledge, and intentions) or have received less attention across major theories of health behavior (e.g., ambiguity and emotions). We also included psychological reactance because this variable can be especially relevant in contexts with government involvement (43)—as was the case during the COVID-19 pandemic—that may lead individuals to feel a threat to their freedom. Thus, we expected that the real-world occurrence of the COVID-19 pandemic would be a fitting context to evaluate this psychological correlate of health behavior.

### Sociodemographic factors and individual level differences relevant to vaccination

Prior research has demonstrated a relationship between general and COVID-19 specific vaccination behavior with various sociodemographic factors (44). For example, in one study conducted with US respondents, individuals who were more vaccine hesitant for COVID-19 were younger and more likely to be female and Black and American Indian or Alaskan Native (45). The direction of the relationship between various sociodemographic factors and vaccination behavior is not always consistent, such as in the case of education or religious affiliation (46).

Various individual level differences may also play a role in health behavior and thus may be relevant to vaccine hesitancy. A meta-analysis of research on health literacy—skills required for people to access, understand, and make use of health information (47) — indicated that health literacy is positively, but weakly, associated with healthier behaviors (48). In another meta-analysis on dispositional optimism, or the tendency to expect outcomes to be good (49), optimism was associated with various physical health outcomes with a small to moderate effect size (50). Another relevant and important construct is tolerance for uncertainty. Although additional research is needed to determine the extent and strength of relationships, tolerance for uncertainty has been associated with medical decision making across several studies (51). In the present study, we assessed these sociodemographic factors and individual level differences because we considered them to also be relevant to vaccination.

### The present research

Although several research studies have examined the relationship among various factors relevant to health behavior and vaccination, further research is needed to include diverse populations. Including diverse populations would lead to a better understanding of what predicts vaccine behavior among different samples, which in turn, would inform strategic approaches to encourage vaccination (e.g., tailoring of health communications). Indeed, researchers concluded from a systematic review of the literature on vaccine hesitancy that most research on this topic is derived from Europe and the Americas (52).

Importantly, the present research extends upon decades of prior work on predictors of vaccine hesitancy by exploring the role of the cultural context. The goal of this research was not to test a specific health behavior theory, but rather to apply what we might expect based on major theories of health behavior to better understand psychological correlates of vaccine hesitancy in a cross-cultural context. Specifically, in the present research, we collected separate samples of American and Israeli participants in order to conduct a cross-cultural comparison of psychological correlates of vaccine hesitancy. Indeed, additional research on psychological correlates of vaccine hesitancy is needed among non-US samples. We use a 2 (Nation: US, Israel) × 2 (Vaccine Status: Vaccinated, Unvaccinated) factorial design. Further, the present research was specifically conducted in the context of the COVID-19 pandemic. However, what is learned in the present context can also provide insight for various other vaccination campaigns as well as during a future pandemic.

### Hypotheses

1. Consistent with various theories of health behavior and research, we hypothesized that unvaccinated individuals (compared with vaccinated individuals) would report higher levels of perceived ambiguity and higher reactance, as well as lower risk perceptions of COVID-19, lower perceived knowledge about COVID-19 and COVID-19 vaccination, and lower intentions to vaccinate.
2. Next, we hypothesized that unvaccinated individuals (compared with vaccinated individuals) would report higher levels of anger and lower levels of fear while thinking about COVID-19 vaccination. Importantly, we included generalized measures of emotion—that is, we did not assess why individuals experienced anger or fear about COVID-19 vaccination—however, we based our hypotheses on possible reasons these emotions may have been experienced. That is, we expected that unvaccinated individuals would report experiencing higher anger in response to government mandates of vaccination, but lower fear about COVID-19 vaccination, consistent with lower risk perceptions from Hypothesis 1 (e.g., “COVID-19 is not severe and so there is no reason to feel fear from the COVID-19 vaccine as it’s unnecessary”).
3. We treated analyses regarding positive emotions as exploratory due to less research on positive emotions in the literature on health behavior.
4. Further, although we expected to observe cultural differences in the prevalence of psychological correlates of health behaviors across samples, we treated these analyses as exploratory. For example, perceptions of ambiguity or risk may differ among cultures but we did not have specific hypotheses for sample differences relevant to nationality. Rather, we examined differences across cultures to inform culture-specific interventions.

## General methods

### Overview

Data were collected in March–June 2023 from two studies with the same design and measures that separately took place in the United States and Israel. Recruitment occurred for each study simultaneously at Kent State University in the United States and at University of Haifa in Israel. Each university provided IRB approval. Eligibility criteria included being age 18 years or older, a resident of either the United States or Israel, and fluency in either English or Hebrew depending on respective location. Of note, although participants were not directly asked if they were an American or Israeli citizen, they were categorized as American or Israeli depending on their respective university, where they indicated living, and their language fluency. It is possible that non-citizens were incorporated into the sample, although they would likely comprise a small percentage of the overall sample. Analyses concerning relationships of perceived ambiguity with risk perceptions, emotions, intentions, and information seeking are reported elsewhere (72).

### Study design and procedure

Participants were recruited using the SONA system subject pool through the Department of Psychological Sciences at Kent State University and the School of Public Health at University of Haifa. Each study was administered online through Qualtrics as a survey on attitudes and beliefs regarding vaccination. Participants first completed a screener to confirm they were eligible to participate followed by informed consent. After this step, participants completed a survey of measures including vaccination history, perceptions of ambiguity and risk, emotion, behavioral intentions, individual level differences, and demographic items.

### Participants

Data collection occurred until the end of the academic semester across each university. Data were collected from 234 Israeli participants and 336 American participants. Data from 36 participants (6 Americans and 30 Israelis) were then removed due to low responsiveness (i.e., less than half the survey was completed). The final sample for analyses included 330 American and 204 Israeli participants (combined *n* = 534).

### Measures

The survey underwent pilot testing with American and Israeli participants to confirm that participants understood each measure. Of note, all measures were translated from English to Hebrew for use with Israeli participants. To guarantee that measures were translated accurately, 3 bilingual, native Hebrew speakers reviewed the translation of measures. Measures that are relevant to hypotheses are reported below.

#### Vaccination status

*Vaccination status* was assessed with one item (created for this study): “Are you fully vaccinated against COVID-19, which means that you received either a single-dose vaccine (such as Johnson & Johnson) or a two-dose series vaccine such as Pfizer or Moderna? The names of authorized vaccines include: Pfizer, AstraZeneca, Moderna, Johnson & Johnson, Sinopharm BIBP, CoronaVac, Covaxin, Novavax, Convidecia, and Sputnik V” (coded as 0 = No, 1 = Yes). Participants were also given the option to respond “Do not know,” and those who did were recoded as missing data for this item (this only applied to 3 Israeli participants).

#### Psychological correlates

Three measures of *risk perception* were used: perceived susceptibility (adapted from (53); average of 3 items; α_American_ = 0.77, α_Israeli_ = 0.59), worry (adapted from (53, 54); average of 3 items; α_American_ = 0.95, α_Israeli_ = 0.96), and perceived severity (adapted from (55); average of 12 items; α_American_ = 0.90, α_Israeli_ = 0.88). Items assessing *perceived susceptibility* were: “I feel very vulnerable to being infected with COVID-19 in the next year” (1 = strongly disagree to 7 = strongly agree), “Overall, how likely is it that *you* will be infected with COVID-19 in the next year?” (1 = extremely unlikely to 7 = extremely likely) and “Overall, how do you think *your* chance of being infected with COVID-19 in the next year compares to other [women/men] of your age in [United States/Israel]?” Items assessing *worry* were: “How [anxious are you/much do you worry/much are you concerned/about being infected with COVID-19?” (1 = not at all to 7 = a lot)]. Finally, items assessing *perceived severity* were: “COVID-19 is [a serious condition/ dangerous/ life-threatening] for [immunocompromised individuals/older adult individuals/young adults/children] without any pre-existing health conditions”” (1 = strongly disagree to 4 = strongly agree). For the perceived severity items, participants could select “do not know,” and those who did were recoded as missing data (no participants responded “do not know” for this measure).

*Perceived ambiguity* (adapted from (56, 57); average of 3 items; α_American_ = 0.80, α_Israeli_ = 0.82) was assessed with, “There are many limitations of the existing information about COVID-19 vaccines,” “There is a lot that is unknown about COVID-19 vaccines” and “Leading scientists and experts have conflicting opinions about COVID-19 vaccines” (1 = strongly disagree, 4 = strongly agree).

*Emotions* (58) included subscales for *fear* (average of 4 items; α_American_ = 0.90, α_Israeli_ = 0.89), *anger* (average of 4 items; α_American_ = 0.92, α_Israeli_ = 0.95), *happiness* (average of 4 items; α_American_ = 0.89, α_Israeli_ = 0.83), and *relaxed* (average taken from 4 items; α_American_ = 0.89, α_Israeli_ = 0.85). In this measure, participants were asked to indicate the extent to which they experience specific affective states when they think about COVID-19 vaccination (1 = not at all to 7 = an extreme amount). Items used to assess fear include “fear,” “worry,” “panic,” and “scared.” Items used to assess anger include “anger,” “rage,” “mad,” and “pissed off.” Items used to assess happiness include “happy,” “enjoyment,” “satisfaction,” and “liking.” Finally, items used to assess relaxation include: “calm,” “relaxation,” “chilled out,” and “easygoing.”

Two separate items were used to assess perceived knowledge: *perceived knowledge about COVID-19* (59) and *perceived knowledge about COVID-19 vaccination* [modified from (59)]. The former was assessed with: “Overall, how would you rate your level of knowledge about COVID-19 (for example, what it is, how it is transmitted, how to protect yourself, etc.)?” and the latter was assessed with: “Overall, how would you rate your level of knowledge about COVID-19 vaccination (for example, what options there are, what are the benefits and side effects, etc.)?” (1 = no knowledge at all to 4 = a lot of knowledge; 5 = do not know). Responses of “do not know” were again coded as missing data (for the perceived knowledge about COVID-19 item, this only applied to 10 American participants and 7 Israeli participants, and for the perceived knowledge about COVID-19 vaccination item, this only applied to 9 American participants and 9 Israeli participants.

*Reactance* [adapted from (60); average of 3 items; α_American_ = 92, α_Israeli_ = 87] included items such as “Vaccination recommendations from the government annoy me” and “The government is trying to manipulate me with these vaccination recommendations” (1 = strongly disagree; 5 = strongly agree).

*Intentions* (created for this study) consisted of a single item: “I intend to get vaccinated against COVID-19 at some point in the future” (1 = strongly disagree; 4 = strongly agree).

#### Sociodemographic factors and individual level differences

Standard demographic items were measured: *age* (continuous), *gender* (coded as 0 = female, 1 = male), *year in school* (coded as 1 = freshman, 2 = sophomore, 3 = junior, 4 = senior, and 5 = graduate student), *race* (in the American sample only; coded as 0 = non-white, 1 = white) and *religious affiliation* (in the Israeli sample only; coded as 0 = non-Jewish, 1 = Jewish). Participants also reported individual level differences. *Tolerance for ambiguity* (61) was assessed as the average of 6 items (1 = strongly disagree to 4 = strongly agree; α_American_ = 0.73, α_Israeli_ = 0.76). In this measure, participants were asked to imagine they are “considering having a medical test that checks for cancer,” but conflicting opinions about the test exist. They were then asked to rate their agreement with statements such as “I would be afraid of trying the test” (1 = strongly disagree, 4 = strongly agree). *Health literacy* (62) was assessed as the average of 3 items, such as “How often do you have problems learning about a medical condition because of difficulty understanding written information?” (1 = none of the time to 5 = all of the time; α_American_ = 0.57, α_Israeli_ = 0.23). Finally, *dispositional optimism* (63) was assessed as the average of 6 items, such as “I rarely count on good things happening to me” (scored so that higher values indicate greater optimism; 1 = strongly disagree to 5 = strongly agree; α_American_ = 0.85, α_Israeli_ = 0.65).

### Overview of analyses

All analyses were conducted in IBM SPSS Statistics Version 27. First, we conducted chi square tests and ANOVAs (depending on whether the dependent measure was a categorical or continuous variable), to determine whether American and Israeli participants differed on any sociodemographic and individual level difference variables. Sociodemographic and individual level difference variables that were not equally distributed across groups were then controlled for in subsequent ANCOVAs that were used to test hypotheses. Next, 12 ANCOVAs controlling for gender, age, education, health literacy, and dispositional optimism were conducted to test main effects of nation and vaccine status, and their interaction, across various psychological correlates of health behavior including: health cognitions (perceived ambiguity, perceived susceptibility, worry, perceived severity, perceived knowledge about COVID-19, perceived knowledge about COVID-19 vaccination, and reactance), emotion (fear, anger, happiness and relaxation), and vaccination intentions.

## Results

See Table 1 for bivariate correlations among study variables. The nature of these correlations was generally consistent with expectations. For example, individuals who reported higher worry about COVID-19, perceived higher severity of COVID-19, and higher knowledge about COVID-19 and COVID-19 vaccines also reported higher intentions to vaccinate. Further, individuals who experienced higher reactance reported lower intentions to vaccinate. With respect to variables that are not generally considered in major theories of health behavior, such as perceived ambiguity and emotion, we note that people who perceived higher ambiguity about COVID-19 vaccines also perceived COVID-19 to be less severe, reported lower knowledge about COVID-19 vaccines, experienced higher reactance, had lower intentions to vaccinate, and also reported higher fear, lower happiness, and lower relaxation. In the interest of brevity, we do not provide an extensive discussion of all correlations, because these analyses were secondary to the others reported and discussed in more detail.

**Table 1:** Bivariate correlations among study variables.

	1	2	3	4	5	6	7	8	9	10	11	12
1. Perceived ambiguity	–	0.01	−0.07	−0.17**	−0.05	−0.23**	0.46**	0.31**	0.28	−0.37**	−0.31**	−0.37**
2. Perceived susceptibility		–	0.41**	0.13**	0.01	0.04	−0.06	0.02	−0.02	−0.03	−0.07	0.08
3. Worry			–	0.26**	0.04	0.08**	−0.09*	0.17**	0.06	0.12	0.02	0.18**
4. Perceived Severity				–	0.09	0.12*	−0.37**	−0.11**	−0.24**	0.27**	0.24**	0.48**
5. Perceived COVID-19 knowledge					–	0.48**	−0.17**	−0.06	−0.03	0.15**	0.12**	0.15**
6. Perceived COVID-19 vaccine knowledge						–	−0.22**	−0.05	0.01	0.24**	0.23**	0.22**
7. Reactance							–	0.42**	0.46**	−0.46**	−0.44**	−0.62**
8. Fear								–	0.73**	−0.12**	−0.25**	−0.23**
9. Anger									–	−0.11*	−0.21**	−0.37**
10. Happiness										–	0.72**	0.45**
11. Relaxed											–	0.40**
12. Vaccination intentions												–
*M*	2.78	3.09	2.50	2.89	3.28	2.92	2.53	2.14	1.91	2.24	2.71	2.61
SD	0.69	1.24	1.54	0.55	0.80	0.90	1.14	1.39	1.40	1.35	1.46	0.94
Range	1–4	1–7	1–7	1.25–4	1–5	1–5	1–5	1–7	1–7	1–7	1–7	1–4

*p < 0.05, **p < 0.01.

See Table 2 for the distribution of sociodemographic characteristics and individual level differences among samples. Findings from preliminary chi square tests and ANOVAs demonstrated that the samples of American and Israeli participants differed on several sociodemographic and individual level difference factors: gender [*X*^2^(1) = 18.18, *p* < 0.001], age [*F*(1,533) = 133.64, *p* < 0.001], education [*F*(1,533) = 137.98, *p* < 0.001], health literacy [*F*(1,529) = 20.13, *p* < 0.001], and dispositional optimism [*F*(1,524) = 5.58, *p* = 0.019]. Compared with the Israeli sample, the American sample was younger, had a greater proportion of males (21.5% male in the American sample versus 7.4% male in the Israeli sample), and had a higher level of education (i.e., there were more Americans at a later year in school). Further, compared with the Israeli sample, the American sample self-reported higher health literacy, but lower levels of dispositional optimism. Thus, these factors were controlled for in subsequent ANCOVAs.

**Table 2:** Distribution of sociodemographic characteristics and individual level differences.

Sociodemographic variables				
	U.S. (*n* = 330)		Israel (*n* = 204)	
	*M* (SD)	Range	*M* (SD)	Range
Age (in years)	20.21 (2.34)	18–34	23.45 (4.15)	19–55
	*N*	Percentage %	*N*	Percentage %
Gender				
Female	262	79.4	188	92.6
Male	66	20.0	13	6.4
Race				
White	268	81.5	–	–
Black or African American	28	8.5	–	–
American Indian or Alaska Native	0	0	–	–
Asian or Asian American	14	4.3	–	–
Native Hawaiian or Pacific Islander	0	0	–	–
Middle Eastern or North African	2	0.6	–	–
Other	15	4.6	–	–
I would rather not report this	2	0.6	–	–
Religious affiliation				
Jewish	–	–	108	56.3
Christian	–	–	17	8.9
Muslim	–	–	48	25.0
Druze	–	–	14	7.3
Other	–	–	1	0.5
Atheist	–	–	3	1.6
I would rather not report this	–	-	1	0.5
Education				
Freshman	94	28.5	169	82.8
Sophomore	91	27.6	14	6.9
Junior	84	25.5	16	7.8
Senior	58	17.6	2	1.0
Graduate student	3	0.9	3	1.5

Individual differences				
	U.S. (*n* = 330)		Israel (*n* = 204)	
	*M* (SD)	Range	*M* (SD)	Range
Tolerance for ambiguity	2.50 (0.47)	1–3.67	2.45 (0.53)	1–4
Health literacy	3.86 (0.77)	1.67–5	3.56 (0.68)	2.33–5
Dispositional optimism	3.04 (0.78)	1–5	3.19 (0.60)	1–4.83

Valid percentage was reported to account for missing data. Race was collected only in the American sample, whereas religious affiliation was collected only in the Israeli sample. For this reason, we were not able to check whether these variables differed across samples. Valid data is unavailable for gender among 2 participants in the American sample and 2 participants in the Israeli sample. Gender [*X*^2^(1) = 18.18, *p* < 0.001], age [*F*(1,533) = 133.64, *p* < 0.001], education [*F*(1,533) = 137.98, *p* < 0.001], health literacy [*F*(1,529) = 20.13, *p* < 0.001], and dispositional optimism [*F*(1,524) = 5.58, *p* = 0.019] differed between samples. Tolerance for ambiguity did not significantly differ between samples [*F*(1,524) = 1.448, *p* = 0.229].

See Table 3 for descriptive statistics and Table 4 for inferential statistics from ANCOVAs. When reviewing the main effects of Nation (US, Israel) on various psychological correlates of health behavior, Israeli participants reported higher perceptions of ambiguity, higher worry, higher fear, and higher anger compared to American participants. However, Israeli participants reported lower perceived severity of COVID-19 compared to American participants. There were no significant differences in perceived susceptibility, perceived knowledge about COVID-19 and COVID-19 vaccination, reactance, happiness, relaxation, or vaccination intentions as a function of nation. When reviewing the main effects of Vaccination Status (Vaccinated, Unvaccinated) on various psychological correlates of health behavior, there were significant differences between vaccinated and unvaccinated participants on perceptions of ambiguity, perceptions of susceptibility, perceptions of severity, worry, reactance, fear, anger, happiness, relaxation, and intentions to vaccinate. Of note, significant differences between vaccinated and unvaccinated individuals on these factors were mostly consistent with hypotheses and theoretically expected relationships. Consistent with hypotheses, unvaccinated individuals reported higher perceptions of ambiguity, higher reactance, and higher anger compared to vaccinated participants. Further, unvaccinated individuals reported lower perceptions of susceptibility, lower perceptions of severity, lower worry about getting COVID-19, lower positive emotion (happiness and relaxation), and lower intentions to vaccinate in the future for COVID-19 compared to their vaccinated counterparts. Inconsistent with hypotheses, unvaccinated individuals reported higher fear compared with vaccinated participants. There were no significant differences in perceived knowledge about COVID-19 or COVID-19 vaccination as a function of vaccination status.

**Table 3:** Health cognitions, emotions, and intentions as a function of nation and vaccine status.

	Nation				Vaccine Status			
	U.S. (*n* = 319)		Israel (*n* = 190)		Vaccinated (*n* = 459)		Unvaccinated (*n* = 50)	
Outcome	*M*	*SE*	*M*	*SE*	*M*	*SE*	*M*	*SE*
Health cognitions								
Perceived ambiguity	2.82	0.07	3.12	0.10	2.78	0.03	3.16	0.11
Perceived susceptibility	2.75	0.12	3.11	0.17	3.19	0.06	2.67	0.19
Worry	2.02	0.14	2.57	0.21	2.61	0.07	1.99	0.23
Perceived severity	2.89	0.05	2.60	0.08	2.87	0.03	2.61	0.09
Perceived COVID-19 knowledge	3.25	0.07	3.27	0.10	3.20	0.04	3.32	0.12
Perceived COVID-19 vaccine knowledge	2.92	0.08	2.88	0.11	2.78	0.04	3.01	0.13
Reactance	2.96	0.10	3.28	0.15	2.47	0.05	3.77	0.16
Emotion								
Fear	2.05	0.13	2.99	0.19	2.17	0.07	2.87	0.21
Anger	1.93	0.13	2.95	0.19	1.91	0.07	2.97	0.21
Happiness	1.98	0.13	1.70	0.19	2.29	0.07	1.40	0.21
Relaxed	2.46	0.14	2.06	0.20	2.79	0.07	1.73	0.22
Intentions								
Intentions to vaccinate	2.34	0.08	2.15	0.12	2.62	0.04	1.87	0.13

**Table 4:** Inferential statistics of nation, vaccine status, and their interaction on health cognitions, emotions, and intentions.

	Nation			Vaccine Status			Interaction		
Outcome	*F*	*p-*value	Partial eta squared	*F*	*p-*value	Partial eta squared	*F*	*p-*value	Partial eta squared
Health cognitions									
Perceived ambiguity	6.48	0.011	0.013	12.03	0.001	0.023	0.05	0.821	0.000
Perceived susceptibility	2.88	0.090	0.006	7.00	0.008	0.014	0.02	0.879	0.000
Worry	4.49	0.035	0.009	6.86	0.009	0.014	0.18	0.669	0.000
Perceived severity	11.49	<0.001	0.022	13.31	<0.001	0.026	0.33	0.563	0.001
Perceived COVID-19 knowledge	0.02	0.888	0.000	0.99	0.321	0.002	1.45	0.230	0.003
Perceived COVID-19 vaccine knowledge	0.09	0.770	0.000	3.03	0.083	0.006	1.45	0.230	0.003
Reactance	2.94	0.087	0.006	58.97	0.000	0.105	2.66	0.104	0.005
Emotion									
Fear	16.05	0.000	0.031	10.55	0.001	0.021	1.53	0.217	0.003
Anger	18.69	0.000	0.036	24.36	0.000	0.046	2.26	0.133	0.004
Happiness	1.42	0.234	0.003	16.76	0.000	0.032	0.00	0.998	0.000
Relaxed	2.50	0.114	0.005	20.81	0.000	0.041	0.66	0.418	0.001
Intentions									
Intentions to vaccinate	1.58	0.209	0.003	30.20	0.000	0.057	6.51	0.011	0.013

Due to missing data across variables, df of *F* statistics varied (1–2, 495–506).

**Figure 1:**
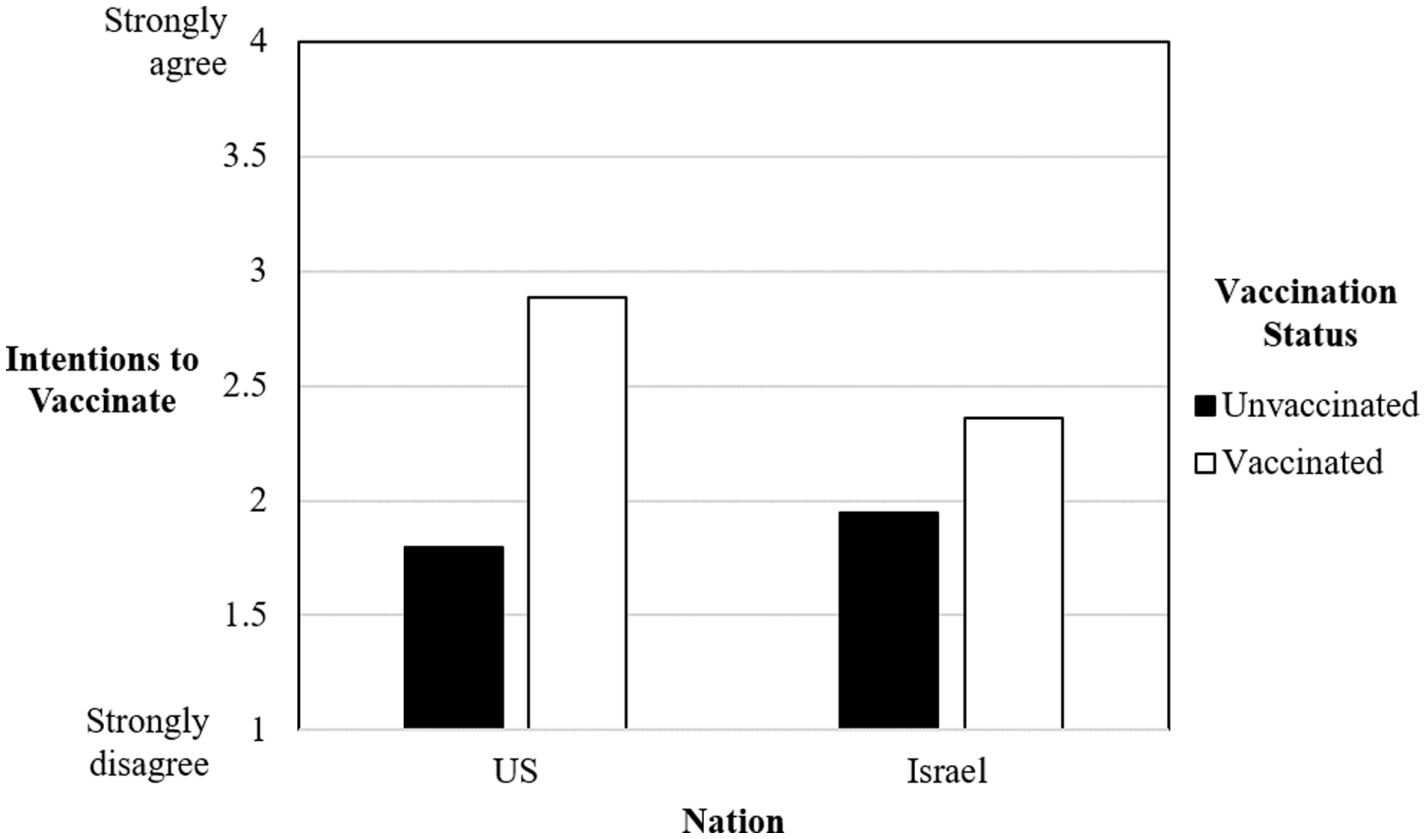
Interaction between nation and vaccination status on intentions to vaccinate.

There was only one significant interaction between nation and vaccination status, and this was in regard to intentions to vaccinate. Vaccinated Americans reported higher intentions to vaccinate (*M* = 2.89, SE = 0.08) compared to vaccinated Israelis (*M* = 2.36, SE = 0.08). However, unvaccinated Americans reported lower intentions to vaccinate (*M* = 1.80, SE = 0.15) compared to unvaccinated Israelis (*M* = 1.95, SE = 0.21). See Figure 1 for a depiction of this relationship.

## Discussion

Vaccine hesitancy is considered one of the top threats to global health (64). Thus, it is important to better understand vaccine hesitant individuals to appropriately intervene. Beyond a broad need to better understand vaccine hesitancy, there is a more immediate need to understand cultural differences and tailor behavioral health interventions appropriately. In the present study, we examined psychological correlates of vaccine hesitancy in a cross-cultural sample of Americans and Israelis. Although findings across samples were mostly consistent with hypotheses and theory, there were differences across cultures.

### Psychological correlates of vaccine hesitancy

Compared with participants who self-reported that they were vaccinated, those who self-reported that they were not vaccinated also reported in the present research that they perceived higher ambiguity about the COVID-19 vaccines, felt higher negative emotion and lower positive emotion about the COVID-19 vaccines, experienced higher reactance about vaccination recommendations, thought they were less susceptible to be infected with COVID-19, thought that getting COVID-19 was less severe, felt less worry about getting COVID-19, and indicated lower intentions to receive a future COVID-19 vaccination. Overall, findings are consistent with how these psychological correlates of health behavior are expected to operate across various theoretical frameworks. These results are also mostly consistent with hypotheses, with the exception of one finding. Inconsistent with the hypothesis that unvaccinated individuals would report lower fear when thinking about COVID-19 vaccines compared with vaccinated individuals, we instead found that unvaccinated individuals reported higher fear. This result may have occurred if unvaccinated individuals were unvaccinated in part due to fear of the vaccines themselves.

Overall, a different profile of thoughts and feelings between vaccinated and unvaccinated individuals emerged. Importantly, findings provide concrete areas for intervention when it comes to vaccine hesitancy. That is, public health officials and practitioners might focus on changing these thoughts and feelings of unvaccinated individuals to match those held by vaccinated individuals. For example, focusing on decreasing perceptions of ambiguity with “normalization of uncertainty” interventions [e.g., (20, 65)] may prove useful. Further, applying a self-affirmation intervention can reduce the effects of reactance [e.g., (66)]. Indeed, the present research provides tangible areas to focus on for vaccination interventions.

Despite several differences, vaccinated and unvaccinated participants did not differ in their perceptions of knowledge about COVID-19 and COVID-19 vaccination. It is possible that the timing of data collection and the saturation of information about COVID-19 led to similar levels of perceived knowledge among vaccinated and unvaccinated individuals across both countries. More specifically, the pandemic had been ongoing for several years at the time of data collection. Further, technology had increased the availability and overwhelm of several voices on COVID-19, referred to as a “saturation” effect (67). In turn, people across countries may have generally felt knowledgeable about COVID-19 and COVID-19 vaccination. Indeed, the average responses to the perceived knowledge measures were above the midpoint for both American and Israeli participants.

### Cross-cultural differences

Israeli participants reported higher perceptions of ambiguity about COVID-19 vaccines, higher fear and higher anger about COVID-19 vaccines, as well as higher worry about COVID-19, compared to American participants. Further, Israeli participants reported lower perceptions of COVID-19 severity compared to American participants. Although exact reasons for these cultural level differences is unknown, these differences underscore the need to examine health behavior within a cultural context. One interpretation for these cultural differences may be related to politicization of the vaccine. Indeed, the vaccines were highly politicized in both countries, but there was stronger involvement of authorities and limits to freedom in Israel during the vaccine rollout. COVID-19 vaccines were disseminated in Israel earlier than other countries due to a deal with Pfizer that allowed Pfizer to collect individuals’ personal vaccine data. Additionally, this deal also made Pfizer the dominant vaccine available in Israel, compared with more options that were available in the United States and elsewhere around the world. The United States and much of the world followed the data on vaccine efficacy and rollout from Israel’s vaccination campaign (73). This arrangement and subsequent debates among Israeli experts regarding vaccination policy may have created more vaccine reluctance and hesitation among the Israeli sample (68). Further, Israel also offered a “green pass” incentive at a federal level to allow people who were fully vaccinated, participating in a vaccine trial, or who recently recovered from COVID-19 to have access to locations that were otherwise closed off to prevent the spread of infection [i.e., businesses; (69)]. This restriction on movement at a federal level, although done to help prevent spread of infection, may have contributed to more negative sentiment among the Israeli sample.

Beyond the political context, several additional factors may help explain the observed differences between Israeli and American participants. First, differences in socio-cultural characteristics may have played a role. Israel’s more collectivist society with dense social networks (74) may have amplified the spread of both emotions and opinions about vaccination among community members. In contrast, a stronger emphasis on individualism and personal rights in the United States (75) may have led to more varied reactions to COVID-19 vaccines. Second, differences in media environment may have been important. Israel’s small size may have facilitated more uniform media coverage of vaccination issues through mainstream channels. The more diverse media landscape and multiple information sources in the United States (see for example (76))—including the prevalence of social media as a source of COVID-19 news (77)—and the polarization of trust in various news outlets (78) may have created less unified social norms around vaccination, allowing for a broader range of perspectives and potentially influencing emotional responses. Third, Israel’s centralized healthcare system, while enabling rapid and uniform policy implementation, may have intensified feelings of institutional control. The decentralized U.S. system allowed for more flexibility in vaccine rollout and policy enforcement, potentially reducing feelings of systemic pressure. Fourth, the historical context between countries may also explain differences in results. Israel’s previous experience with national emergencies may have influenced both institutional trust and emotional responses to government mandates. The United States has a longer history of vaccine debates predating COVID-19 (79), which may have positioned the COVID-19 vaccine within an existing framework of vaccine attitudes. Finally, demographic differences in average age, population density, and family structure between the countries may have influenced risk perception and vaccine attitudes. Additionally, varying levels of ethnic and cultural diversity between the nations may have affected institutional trust and response to health policies.

Indeed, paying greater attention to cross-cultural differences in vaccine hesitancy can allow the respective health ministries in each country to then frame health communications based on the needs of their respective populations. Future researchers may even consider whether these higher perceptions of ambiguity, fear, and anger about COVID-19 vaccines or lower perceptions of COVID-19 severity may have had an unintended spillover effect among Israelis toward other vaccines and vaccine-preventable illnesses.

#### Interaction effect

In the present research, whether individuals were vaccinated or not was associated with whether individuals also reported they were interested in receiving a future COVID-19 vaccination. First, vaccinated Americans reported higher intentions to receive a COVID-19 vaccine in the future compared to vaccinated Israelis. Second, unvaccinated Americans reported lower intentions to vaccinate in the future compared to unvaccinated Israelis. Thus, again, it is important to examine how health behavior might differ in different cultural contexts.

### Limitations

There are limitations of the present research. First, we recruited convenience samples from universities, and the samples are not nationally representative. Indeed, as demonstrated by Table 2, most of the participants in the present research were young adult females, with limited diversity in gender and age. Indeed, males and females differ in COVID-19 risk perceptions and vaccine hesitancy, such that females perceive higher COVID-19 risk, but also tend to be more hesitant toward vaccination (70). Future research should include a more diverse sample in terms of age and gender, and also consider factors such as socioeconomic status. Second, there were also more vaccinated than unvaccinated participants, and future research should increase efforts to recruit unvaccinated participants. Third, data were collected while the pandemic was ongoing for several years (i.e., March–June 2023) rather than at the start of the pandemic in March 2020. Thus, associations among variables may have been weaker than at the start of the pandemic. Fourth, we cannot identify why certain cross-cultural differences exist, as this requires more in-depth research. Indeed, qualitative work may be beneficial for interviewing individuals and isolating themes relevant to their vaccination decisions. Fourth, we examined emotions about COVID-19 vaccination generally, and the exact target of negative and positive emotions (i.e., *why* participants experienced the specific emotions in regard to COVID-19 vaccination) was not examined. Future research should delve further into understanding these emotions. Additionally, the intentions item was written to be broad to apply to both vaccinated and unvaccinated individuals’ experiences (i.e., “I intend to get vaccinated against COVID-19 at some point in the future”). Thus, it is unknown how participants specifically interpreted this measure. Indeed, individuals may have a difference in opinion regarding the primary vaccination vs. additional booster shots, and this may have been reflected in their answers. Future research might consider these differences.

## Conclusion

In the present research, we use cross-sectional data to provide a description of public perceptions about COVID-19 vaccination among vaccinated and unvaccinated Israeli and American adults. Findings revealed cross-cultural differences between the United States and Israel on psychological correlates of vaccine hesitancy. Continued cross-cultural research is necessary for increased vaccination efforts through tailoring of vaccination materials.

## Data Availability

The raw data supporting the conclusions of this article will be made available by the authors, without undue reservation.
