# Corrigendum: Examining psychological correlates of vaccine hesitancy: a comparative study between the US and Israel

**DOI:** 10.3389/fpubh.2025.1619192

**Published:** 2025-06-19

**Authors:** Nicolle Simonovic, Anat Gesser-Edelsburg, Jennifer M. Taber

**Affiliations:** ^1^The Health and Risk Communication Lab, School of Public Health, University of Haifa, Haifa, Israel; ^2^Department of Psychological Sciences, Kent State University, Kent, OH, United States

**Keywords:** vaccine hesitancy, health behavior, risk perception, emotions, ambiguity, intentions

In the published article, there was an error. In the Abstract, we reported results that were exploratory along with results that were consistent with hypotheses, without distinguishing between the two.

A correction has been made to the Abstract. This sentence previously stated:

“Consistent with hypotheses, unvaccinated (vs. vaccinated) individuals reported higher perceived ambiguity, reactance, and anger as well as perceived lower susceptibility, severity, worry, positive emotion, and intentions to vaccinate.”

The corrected sentence appears below:

“Consistent with hypotheses, unvaccinated (vs. vaccinated) individuals reported higher perceived ambiguity, reactance, and anger as well as perceived lower susceptibility, severity, worry, and intentions to vaccinate. Unvaccinated (vs. vaccinated) individuals also reported lower positive emotion.”

The authors apologize for this error and state that this does not change the scientific conclusions of the article in any way. The original article has been updated.

